# Ancient DNA preservation in the Caribbean

**DOI:** 10.1098/rsos.260217

**Published:** 2026-09-09

**Authors:** Jazmín Ramos-Madrigal, Miren Iraeta-Orbegozo, Kathrin Nägele, Maria A. Nieves-Colón, Marcela Sandoval-Velasco, Dorothea Mylopotamitaki, Yadira Chinique de Armas, Jason E. Laffoon, Zara Ali, Carlos Arredondo Antúnez, Robert S. Carr, Patrice Courtaud, Jane S. Day, William Pestle, Armando Rangel Rivero, Thomas Romon, William C. Schaffer, Peter Siegel, Anne C. Stone, Carsten Wiuf, Morten E. Allentoft, Hannes Schroeder

**Affiliations:** ^1^ Globe Institute, Faculty of Health and Medical Sciences, University of Copenhagen, Copenhagen, Denmark; ^2^ School of Archaeology, Faculty of Humanities, University of Copenhagen, Copenhagen, Denmark; ^3^ Department of Mathematical Sciences, Faculty of Science, University of Copenhagen, Copenhagen, Denmark; ^4^ Max-Planck-Institute for Evolutionary Anthropology, Leipzig, Germany; ^5^ School of Human Evolution and Social Change, Arizona State University, Tempe, AZ, USA; ^6^ Department of Anthropology, University of Minnesota Twin Cities, Minneapolis, MN, USA; ^7^ Centro de Ciencias Genómicas, Universidad Nacional Autónoma de México, Mexico City, Ciudad de México, Mexico; ^8^ Department of Anthropology, The University of Winnipeg, Winnipeg, Manitoba, Canada; ^9^ Faculty of Archaeology, Leiden University, Leiden, The Netherlands; ^10^ Museo Antropológico Montané, Universidad de la Habana, Havana, Cuba; ^11^ Archaeological and Historical Conservancy Inc., Davie, FL, USA; ^12^ PACEA, UMR 5199 CNRS, Université de Bordeaux, Pessac, Talence, France; ^13^ Research Atlantica Inc., Boca Raton, FL, USA; ^14^ Kelsey Museum of Archaeology, University of Michigan, Ann Arbor, MI, USA; ^15^ Liberal Arts Department, Phoenix College, Phoenix, AZ, USA; ^16^ Department of Anthropology, Montclair State University, Montclair, NJ, USA; ^17^ Trace and Environmental DNA (TrEnD) Laboratory, School of Molecular and Life Sciences, Curtin University, Perth, Australia

**Keywords:** ancient DNA, bioarchaeology, diagenesis, palaeogenomics, tropics

## Abstract

Ancient DNA (aDNA) analysis has transformed how we study human migration, species dispersals and environmental change, but poor preservation remains a major challenge. This is especially true in tropical settings, where hot and humid conditions accelerate DNA decay. Because aDNA work is destructive, knowing how long DNA survives and under what conditions is crucial for selecting samples likely to yield meaningful data while minimizing damage. Here, we analyse low-coverage shotgun data from over 300 human samples from 33 archaeological sites (3300–400 cal. BP) in the Caribbean to evaluate the effects of sample age, sample type and burial context on DNA preservation. Preservation varied markedly both between and within sites, with sample type and burial context exerting strong effects. Macroscopic inspection, though imperfect, proved a useful first line predictor of molecular preservation. Decay-rate modelling indicates that DNA decays roughly 4× faster in the Caribbean than in temperate regions and about 14× faster than in subpolar settings. Under these conditions we predict that recovery from remains significantly older than approximately 5000 years will be challenging. Encouragingly, this window encompasses most of the region's human history, indicating that much of it remains accessible to aDNA analysis, provided suitable samples can be found and appropriate excavation and post-excavation protocols are followed.

## 1. Introduction

Ancient DNA (aDNA) has yielded important insights into the biogeography and human population history of the Caribbean [1–10]. Yet, in this region, the likelihood of recovering DNA from archaeological samples is generally low owing to poor preservation. DNA tends to preserve better in colder environments than in warmer ones, such as the Caribbean, where mean annual daytime temperatures rarely fall below 25°C [11–14]. Although temperature is a key factor in DNA decay, preservation is also influenced by a range of additional variables that may differ not only between sites but also within a single site or archaeological layer [15]. For instance, water promotes hydrolysis of the phosphodiester bonds in the DNA backbone, producing miscoding lesions and ultimately fragmenting DNA [16,17]. Microorganisms further accelerate degradation by breaking down tissues and releasing nucleases, converting organic material into microbial biomass over time [18]. Soil chemistry also matters, with neutral to slightly alkaline conditions generally favouring DNA survival [19–21].

DNA preservation can be assessed in several ways, most commonly by measuring the endogenous DNA content (i.e. the proportion of DNA derived from the target organism, e.g. human, relative to microbial contamination), the average DNA fragment length and the amount of post-mortem DNA damage caused by cytosine deamination. DNA preservation is influenced not only by environmental conditions but also by the sampled skeletal element. The dense inner ear region of the petrous bone, particularly the cochlea, consistently yields the highest levels of endogenous DNA and is, therefore, often the preferred sampling target [22–24]. More recently, the auditory ossicles have also been shown to preserve DNA at similarly high levels while requiring less destructive sampling than the petrous bone [25]. Teeth likewise tend to preserve DNA better than many other skeletal elements, such as ribs or long bones, and offer the added advantage of harbouring potential pathogen DNA [26]. Within teeth, human endogenous content tends to be higher in the thin cementum layer [24,27], which can be sampled in a minimally invasive way by exposing the outer root surface to lysis buffer while leaving the tooth intact [28]. Overall, the petrous bone, ossicles, and tooth cementum provide the most reliable sources of ancient human DNA, whereas spongy postcranial bone (e.g. ribs) generally yields much lower endogenous content [29].

In addition to sample type, burial setting also influences DNA preservation, as ambient temperature and other microenvironmental factors—including water exposure, soil acidity and microbial activity—can vary substantially [30]. In the Caribbean, skeletal remains are typically recovered from three main settings: open sites, caves and semi-open rock shelters, with occasional finds in water-filled sinkholes representing rare exceptions [2]. Previous studies have reported higher amplification success from cave contexts [31,32], and the only higher-coverage ancient human genome from the region also derives from cave remains in the Bahamas [4]. Nevertheless, the relative contribution of different factors remains difficult to disentangle, and no systematic investigation of DNA preservation in the Caribbean has yet been undertaken. Because aDNA analyses are destructive, there is a strong ethical imperative to target samples likely to yield high information value and to avoid damaging remains unlikely to produce results. Understanding how long DNA survives and under what conditions is, therefore, important not only scientifically but also for minimizing unnecessary damage.

To evaluate how sample age, burial context and sample type shape DNA preservation in tropical environments, we analysed low-coverage shotgun sequencing data from more than 300 human skeletal and dental samples spanning 33 archaeological sites across the Caribbean (figure 1). Here, ‘sample age’ refers to the radiocarbon (^14^C) age of the specimen, expressed in calibrated years before present (cal BP), rather than the biological age-at-death of the individual. To further characterize degradation dynamics, we modelled DNA decay rates for a subset of 75 directly radiocarbon-dated samples with sufficient endogenous human DNA to estimate the fragment-length decay parameter (*λ*), following the analytical framework of Allentoft *et al.* [33]. We additionally compared the shotgun sequencing results with previously published 1240k target-capture data for 82 individuals [5] to assess the efficiency of enrichment in recovering genome-wide information from poorly preserved material. Together, these analyses constitute the first systematic assessment of aDNA preservation in the Caribbean and help guide future sampling strategies and data-generation efforts.

**Figure 1.**
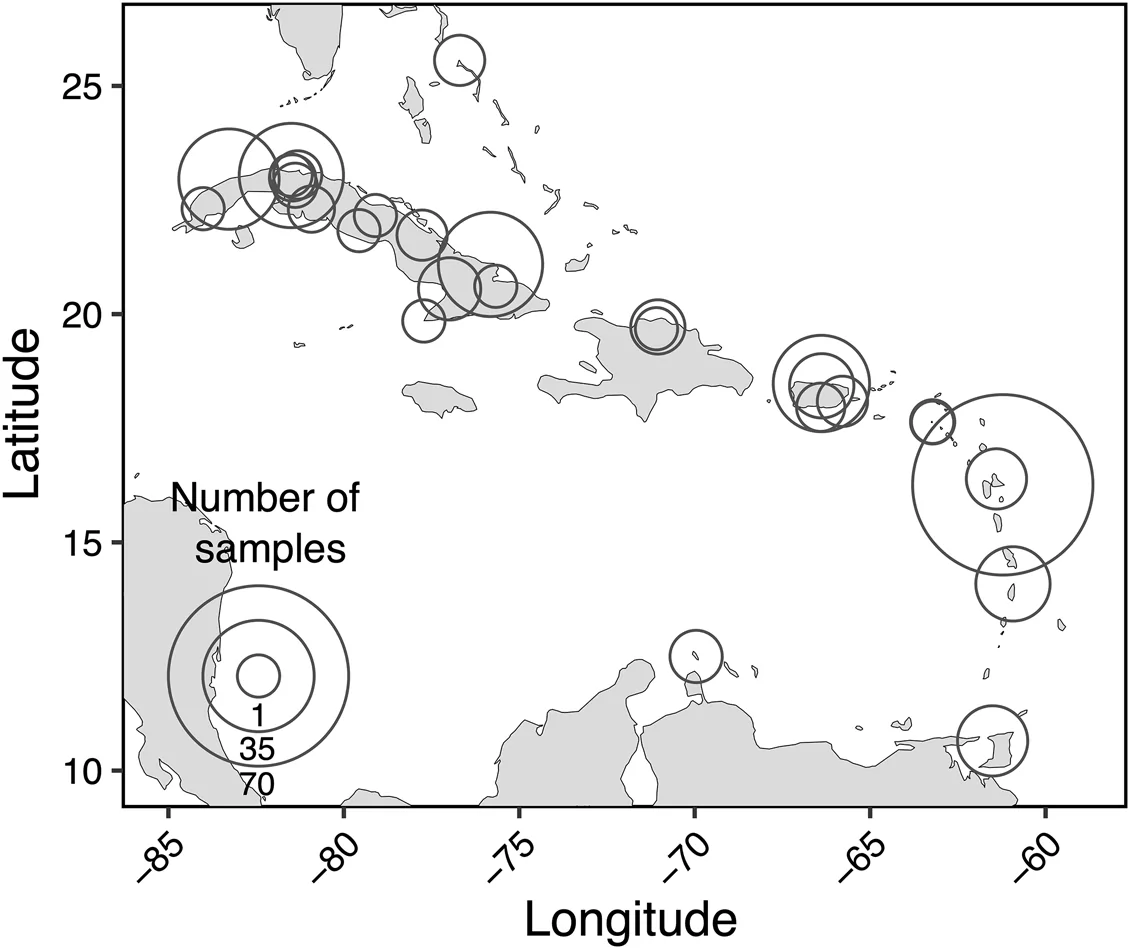
Map of the Caribbean showing the archaeological sites sampled for aDNA. The size of the circles is proportional to the number of human remains sampled for each site.

## 2. Material and methods

### 2.1. Sites and samples

Our dataset comprises low-coverage shotgun sequencing data from 318 human skeletal and dental samples recovered from 33 archaeological sites across the Caribbean and dated to approximately 3200–400 cal BP (figure 1). Before DNA extraction, all samples were visually inspected and classified according to macroscopic preservation as either ‘good’ (e.g. dense and compact) or ‘poor’ (e.g. brittle and chalky). The sample set comprised 217 teeth, 88 petrous bones and 13 rib and long bone fragments recovered from open sites, rock shelters and caves (supplementary material, table S1). Samples were collected between 1998 and 2020, and permission to carry out aDNA analyses and radiocarbon dating was obtained from government agencies and collaborating institutions in the region, following consultation with local archaeologists and other stakeholders (see supplementary material).

### 2.2. Data generation

The samples included in this study were processed across three aDNA laboratories using different extraction and library preparation protocols (supplementary material, table S1). The majority of samples (*n* = 182) were processed in the aDNA laboratory at the University of Copenhagen. DNA was preferentially extracted from tooth cementum [34], while petrous bones were sampled by isolating the cochlea [23]. DNA purification was performed using either silica columns [35] or silica powder with a predigestion step [27]. Libraries were prepared following one of two protocols [36,37]. Additional samples from Cuba, Guadeloupe and Puerto Rico (*n* = 129) were processed at the Max Planck Institute in Jena using silica column-based extractions [38] followed by either double-stranded [36] or automated single-stranded DNA library preparation [39]. The seven remaining samples from Paso del Indio (Puerto Rico) were prepared at Arizona State University using the same silica column-based extraction protocol [38] and standard double-stranded library preparation [36], as described in [40]. Finally, some extracts were subjected to partial UDG treatment, whereas others were left untreated, as detailed in table S1 in the supplementary material. Sequencing was performed on Illumina HiSeq and NextSeq platforms run in single- or paired-end mode. All laboratory work followed established aDNA standards, including physically separated clean rooms, full protective clothing, UV decontamination and stringent negative controls. For full details, see supplementary material.

### 2.3. Data processing

To standardize coverage, datasets with more than 10 million reads were randomly subsampled to approximately 10 million raw reads. For paired-end libraries, only the first read (R1) was processed to ensure comparability of fragment length estimates across experiments. Adapters and low-quality regions were trimmed with AdapterRemoval2 [41], and reads shorter than 30 bp were discarded. The remaining reads were mapped to the human reference genome (GRCh37/hg19) using BWA-backtrack v.0.7.15 [42] with seeding disabled to reduce bias from 5′ deamination, retaining only reads with mapping quality MQ ≥ 30.

For HiSeq4000 data, we further filtered cluster duplicates (i.e. reads mapping to the same position and strand within 5000 bp on the flow cell), retaining only one per cluster [43]. PCR duplicates were removed with Picard tools. Post-mortem DNA damage was quantified with mapDamage 2.0 [44] as the frequency of C→T misincorporations at 5′ read termini (MQ ≥ 30). Contamination was estimated with ContamMix [41] for samples with mtDNA coverage > 2× and hapCon [42] for males with adequate nuclear coverage. Based on these criteria, 20 non-UDG-treated samples with <10% terminal C→T misincorporations and 2 samples with > 10% contamination were excluded (supplementary material, table S2). For more details, see supplemental material.

### 2.4. Assessing DNA preservation

We assessed DNA preservation using three metrics: (i) human endogenous DNA content, calculated as the proportion of trimmed reads ≥30 bp that could be mapped to the human genome with MQ ≥ 30; (ii) cytosine deamination rates, expressed as the frequency of C→T misincorporations at 5′ read termini; and (iii) theoretical median fragment length, estimated by fitting an exponential model in R to the declining portion of the fragment length distribution and taking the reciprocal of the rate parameter (1/*λ*) [33]. This theoretical measure was preferred over the observed read length, which can underestimate fragmentation because very short molecules are typically lost during extraction, library preparation or sequencing. To account for library clonality, all three metrics were calculated both before and after PCR duplicate removal. Post-deduplication results are presented in the main text and figures 2–4, while pre-deduplication values are reported in table S2 in the supplementary material.

**Figure 2.**
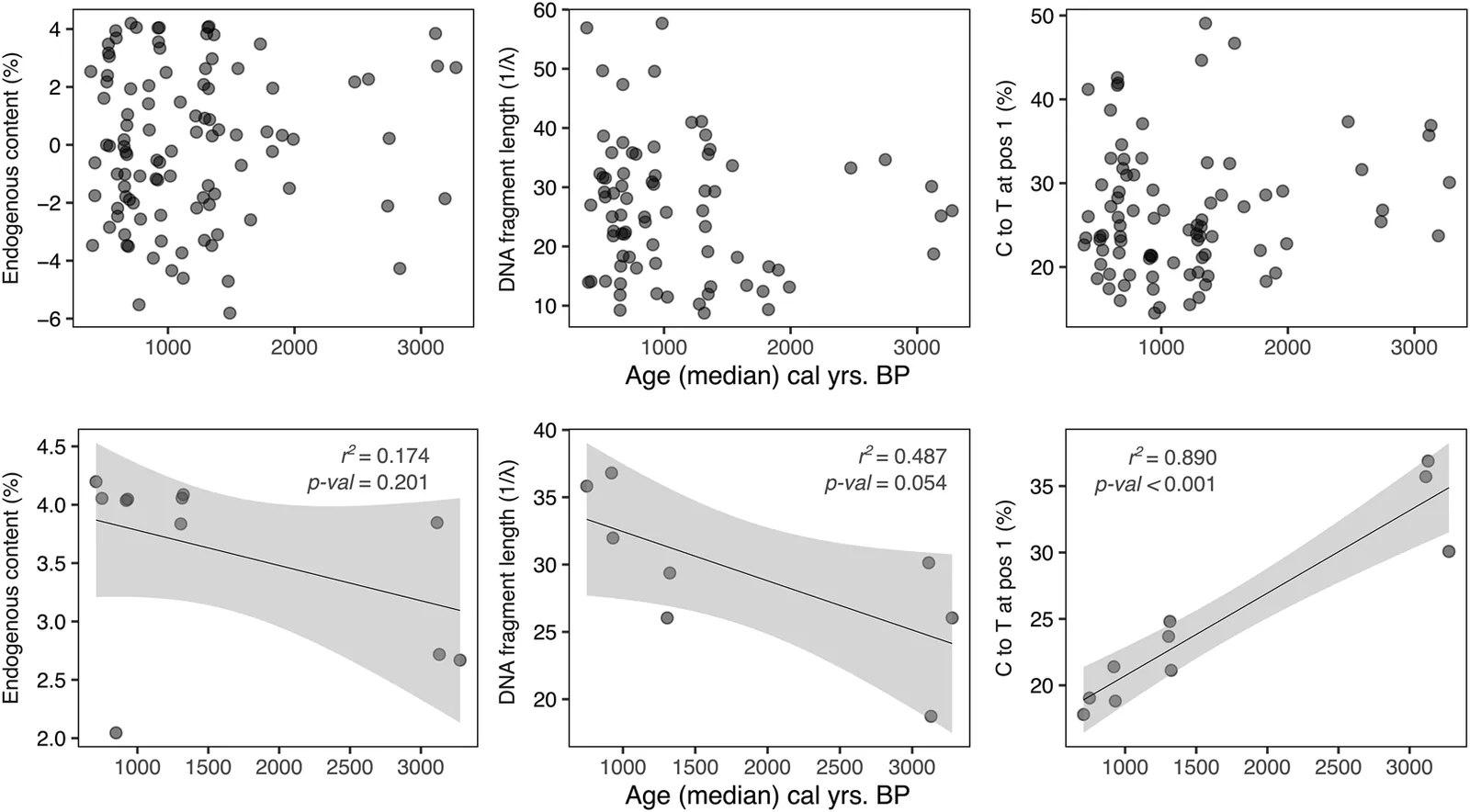
DNA preservation over time. Human endogenous DNA content (%, displayed in log scale), theoretical DNA fragment length (1/*λ*, bp) and C→T misincorporations at 5′ read termini (MQ ≥ 30) for 102 radiocarbon-dated human skeletal samples from 22 archaeological sites in the Caribbean (top) and 11 petrous bones from the site of Canímar Abajo in Cuba only fitted with a linear regression model (bottom). The grey line and shaded area indicate the best fit and corresponding confidence intervals, with the resulting *r*^2^ and *p*-values also shown.

Statistical significance of observed differences was evaluated using two-sample student's *t*-tests in R. Analyses of fragment length and deamination rates were restricted to samples with endogenous content >0.05% (pre-deduplication), and only non-UDG-treated libraries were used for deamination estimates. Categories with fewer than five samples were excluded. Statistical test results for both pre- and post-deduplication datasets are summarized in table S4 in the supplementary material. Importantly, significant differences across sample types and burial settings were consistent between the two datasets. At two sites (Maisabel and Anse à la Gourde), approximately 30% of reads were lost during adapter removal, likely owing to adapter dimer formation; repeating analyses with and without these sites yielded consistent results. We also tested for potential batch effects associated with different library protocols [37,45] and sequencing platforms (NextSeq500, HiSeq2500 and HiSeq4000), but none were detected (see supplementary material).

We also explored multivariate models to assess the joint effects of sample type, burial context and sample age on DNA preservation metrics (endogenous DNA content, fragment length and deamination rates). However, these analyses were limited by the highly unbalanced structure of the dataset and the incomplete availability of key variables, which reduced sample sizes and led to poor model diagnostics. Although some effects (notably sample type and burial context for endogenous DNA content) were detected, they were not consistently reproducible across subsets of the data, indicating sensitivity to data imbalance and confounding. These results suggest that, for this dataset, simpler univariate comparisons provide a more robust and interpretable framework.

### 2.5. DNA decay rates

DNA decay rates were estimated for a subset of 75 directly radiocarbon-dated samples with sufficient endogenous human DNA to estimate the decay parameter (*λ*) from the fragment length distribution, alongside 24 previously published ancient genomes from North America, including Greenland and Alaska [46–52], using the analytical framework of Allentoft *et al.* [33]. To obtain *λ*, we fitted a linear regression to the declining portion of the fragment length distribution of aligned reads (MQ ≥ 30) using the lm function in R and excluding the last five bins. The resulting *λ* values were then used to estimate the decay rate (*k*), as described in Allentoft *et al.* [33] (supplementary material, table S5). To model the expected survival of DNA fragments over time, we also estimated the time (*t*) required for a given proportion (*q*) of fragments of a given length (*L*) (e.g. 99.9% of 30 bp fragments) to undergo at least one strand break, using the following relationship:

$$\begin{array}{r} q=\exp\left(-kLt\right)\;\text{or equivalently},\;t=\frac{-1}{kL}\log\left(q\right). \end{array}$$

### 2.6. Target enrichment (1240k capture)

To assess the efficiency of genome-wide target enrichment (1240k capture), we compared our shotgun sequencing results with previously published 1240k capture data generated on a subset of 82 samples from the region [5]. For these overlapping samples, we quantified the number of 1240k SNPs recovered from shotgun sequencing and retrieved the corresponding SNP counts from the capture dataset [5]. Enrichment factors were calculated as the ratio of target SNPs recovered after capture to those obtained from shotgun sequencing, normalized by the number of trimmed reads.

## 3. Results and discussion

Understanding how long and under what conditions DNA survives is crucial not only for assessing the likelihood of recovery but also for guiding sample selection, helping researchers target remains with higher information potential while avoiding unnecessary damage to Caribbean cultural heritage. In the shotgun sequencing era, endogenous DNA content—the proportion of reads derived from the organism of interest—has become one key screening criterion, as very low values require extensive sequencing and rapidly escalate costs. Endogenous content reflects both natural DNA decay and environmental contamination, processes accelerated by high temperatures and microbial activity [12]. Consistent with these expectations, endogenous human DNA content in our dataset was generally low (median = 0.3%; mean = 5.8%), with most samples (*n* = 192) containing <1% endogenous DNA (supplementary material, table S2).

Only 108 of 318 samples (approx. 34%) exceeded the 1% endogenous threshold typically considered necessary for efficient shotgun sequencing. However, a larger fraction—189 of 318 samples (approx. 59%)—surpassed 0.1%, a commonly used minimum for genome-wide target enrichment (e.g. 1240k capture) [5]. In other words, while relatively few Caribbean samples are suitable for cost-effective shotgun sequencing, more than half of the analysed individuals remain viable candidates for capture-based approaches. These proportions provide a practical baseline for future studies, highlighting both the challenges of working in tropical contexts and the considerable potential to generate informative aDNA datasets from the region.

DNA fragmentation—typically quantified as average fragment length—is a second key indicator of preservation, since shorter molecules reflect progressive strand breakage over time. When fragmentation becomes extreme, very short fragments can no longer be mapped reliably to a reference genome, reducing the number of informative reads. In our dataset, observed fragment lengths averaged around 51 bp, indicating substantial fragmentation, while the corresponding theoretical values derived from the decay constant were shorter at about 25 bp, as expected when accounting for fragments lost during sequencing and mapping. Nevertheless, a substantial fraction of fragments exceeded 30 bp, providing enough mappable reads for downstream analyses.

Cytosine deamination, measured as C→T misincorporations at the 5′ ends of reads, is a third key indicator of preservation. This chemical modification accumulates gradually over time as cytosine residues are converted to uracil and subsequently read as thymine during sequencing [53,54]. In our samples, deamination rates at the first 5′ position averaged around 28%, higher than values reported for samples of similar age from Eurasia (approx. 16–20%) [55–57] but lower than those from Southeast Asia (approx. 34%) [54]. This pattern suggests that hot–humid conditions in Southeast Asia may accelerate deamination more strongly than in the Caribbean. In contrast, fragment lengths were broadly comparable across all three regions [55–58].

In addition to molecular criteria, the macroscopic appearance of skeletal material is often used as a first line indicator of preservation. Hansen *et al.* [24] demonstrated that a simple good/bad visual rating before extraction correctly predicted whether samples would fall above or below a randomly defined 10% endogenous DNA threshold in around 80% of cases. To test whether this holds in the Caribbean, we applied the same good/bad visual rating to our results. Using a cut-off of 1% endogenous DNA to account for the overall poor preservation in the region, visual inspection correctly distinguished samples above or below this threshold in 76% of cases (supplementary material, table S2), with similar accuracy for petrous bones (75%) and teeth (74%). On average, visually well-preserved samples yielded 14% endogenous DNA, compared to just 0.9% in poorly preserved ones. These results suggest that visual inspection offers a practical first-pass strategy, especially valuable where destructive sampling must be minimized and resources for screening are limited.

### 3.1. Ancient DNA preservation over time

Time is a key factor shaping DNA preservation. As molecules break down, fragments become shorter, damage accumulates and endogenous DNA is gradually replaced by microbial DNA from the burial environment. To assess temporal effects in our dataset, we examined endogenous DNA content, theoretical fragment length (1/*λ*) and deamination rates in all directly radiocarbon-dated samples. Across all 102 directly dated individuals, no clear correlation was observed between the radiocarbon age of the samples and any of these metrics (figure 2, top). However, restricting the analysis to 11 petrous bones from a single Cuban site revealed a slight decline in endogenous content and fragment length and a significant increase in deamination rates over time (figure 2, bottom). This suggests that while DNA decay is clearly time dependent, differences between sample types and burial environments can easily mask these trends in bigger datasets. This is consistent with previous studies showing that, even in controlled settings, sample age alone accounts for only a limited proportion (approx. 30–40%) of the variation in DNA preservation [33,59,60].

### 3.2. Ancient DNA preservation by sample type

aDNA preservation varies markedly between skeletal and dental tissues. The inner ear region of the petrous bone is known to preserve DNA better than other skeletal elements, particularly ribs or long bones [22]. To assess this further, we compared endogenous DNA content, theoretical fragment length (1/*λ*) and deamination rates across 199 teeth, 86 petrous bones and 11 rib and long bone fragments (figure 3). Petrous bones yielded significantly higher endogenous DNA content compared to teeth and to ribs and long bones (*p* < 0.01), regardless of the burial context (supplementary material, table S4). Overall, the average human endogenous DNA content in petrous bone was 17.4% compared to 1.2% in teeth and 0.01% in ribs and long bones. Only 6 of the 199 teeth exceeded 10% endogenous DNA, compared to 39 of the 86 petrous bones, whereas all rib and long bone fragments performed poorly, averaging around 0.01% endogenous DNA. Deamination rates and theoretical fragment lengths did not differ significantly between tissue types, with values falling within the range expected for authentic aDNA [53,54]. The only exception was in open sites, where teeth exhibited significantly longer average theoretical fragment lengths (*p* < 0.01) than petrous bones. These findings differ from those reported for Central Asia and Denmark [24], where petrous bones showed higher deamination rates and slightly shorter average fragment lengths than teeth, suggesting more degraded DNA despite higher endogenous content.

**Figure 3.**
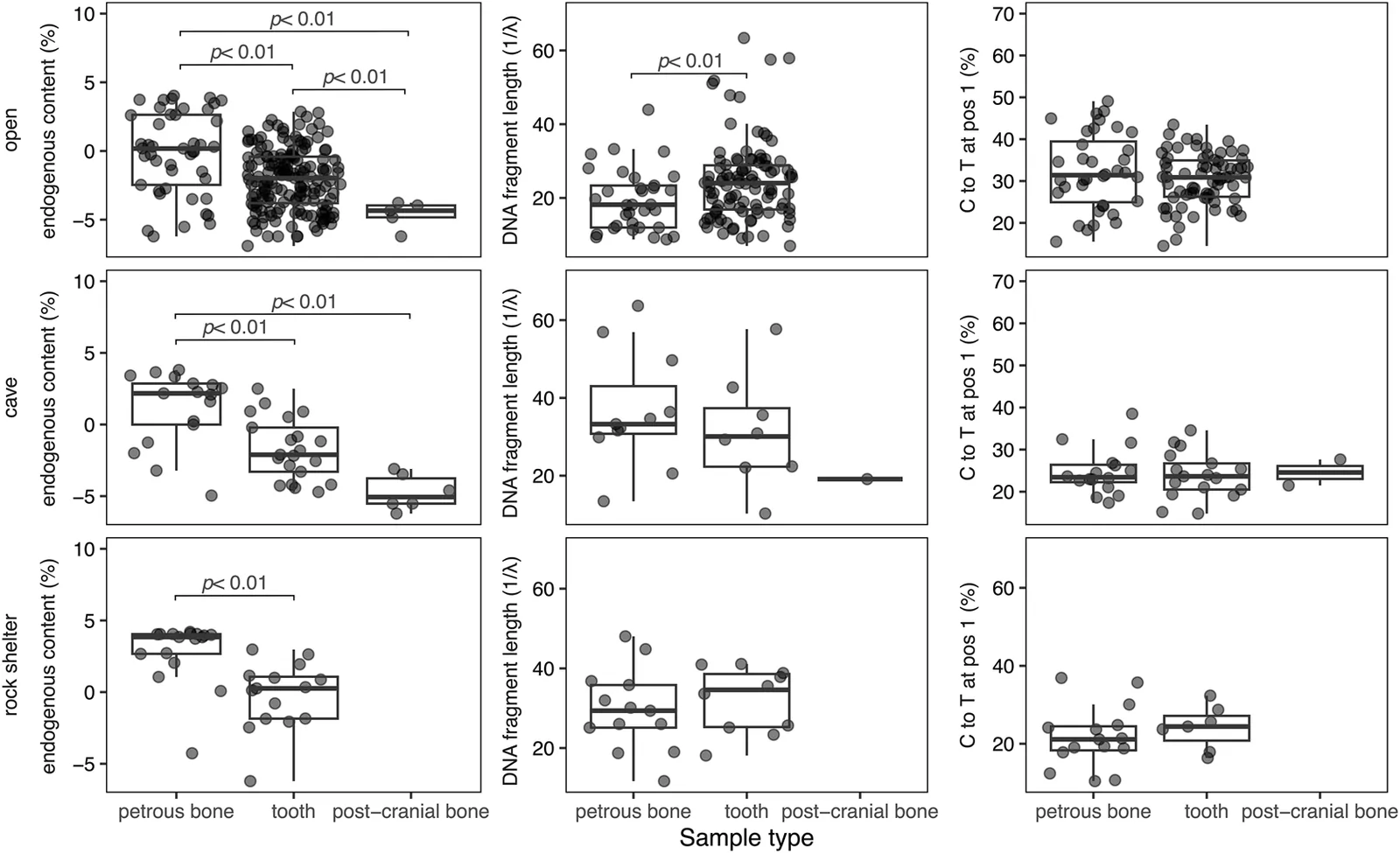
DNA preservation between different sample types across different burial contexts. Human endogenous DNA content (%, displayed in log scale), theoretical DNA fragment length (1/*λ*, bp) and C→T misincorporations at 5′ read termini (MQ ≥ 30) by sample type. Pairwise comparisons with statistically significant differences (*t*-test; *p* < 0.01) are noted at the top.

### 3.3. Ancient DNA preservation by burial context

Human remains in the Caribbean have been recovered from caves, open sites and rock shelters. Caves typically maintain more stable conditions and cooler average temperatures than open sites, and previous studies have suggested that they offer more favourable environments for DNA preservation [4,31,32]. To test this, we compared endogenous content, theoretical median fragment length (1/*λ*) and deamination rates across open sites (*n* = 213), caves (*n* = 44) and rock shelters (*n* = 32) (figure 4; supplementary material, table S4). The human endogenous DNA content was similar between caves and open sites, averaging 5.6 and 2.9%, respectively (*p* > 0.01), but significantly higher in petrous bones from rock shelters (*p* < 0.01), driven largely by exceptional preservation at Canímar Abajo (supplementary material, table S2). Theoretical fragment lengths and deamination rates also varied by burial context: open-site samples had shorter fragments, averaging 23.6 bp, and higher deamination rates of about 31%, than those from caves (33.6 bp, 24%) and rock shelters (30.6 bp, 23%) (figure 4), indicating that caves and rock shelters provide more favourable conditions for DNA preservation, consistent with previous reports [4,32].

**Figure 4.**
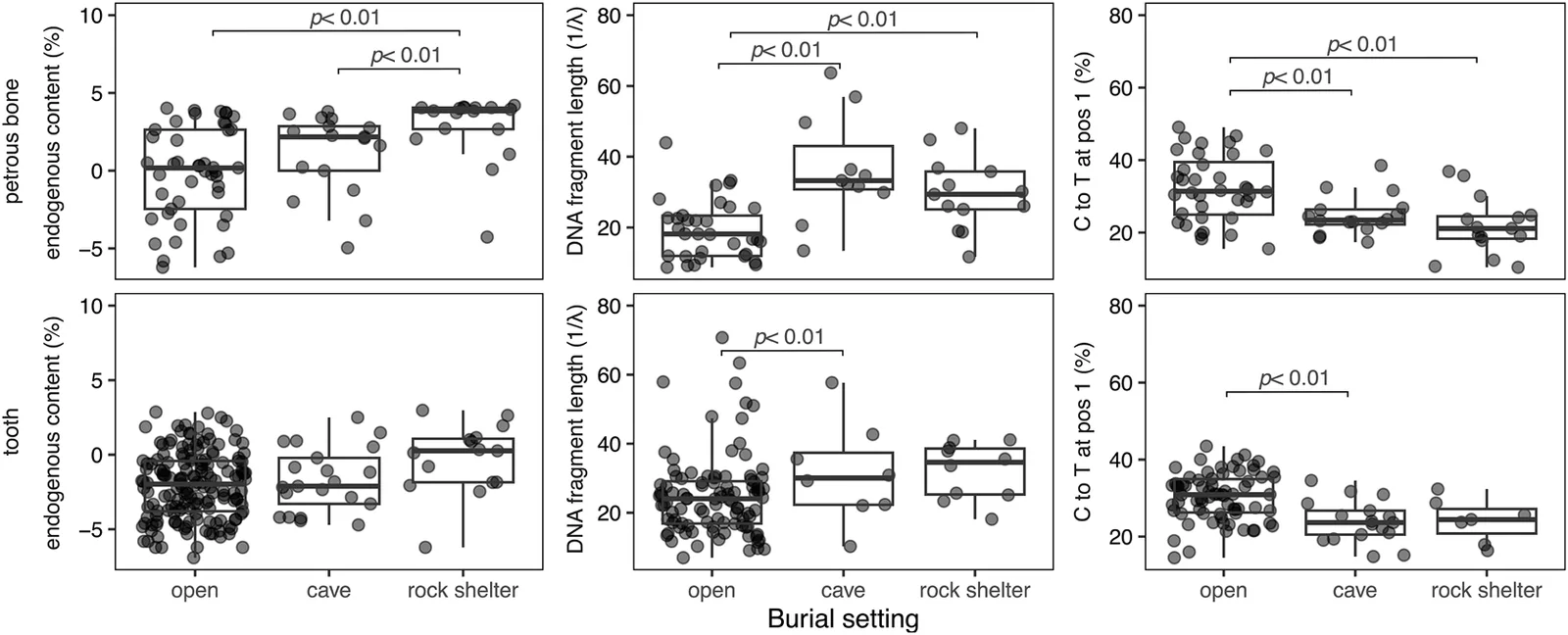
DNA preservation in three distinct burial contexts across different sample types. Human endogenous DNA content (%, displayed in log scale), theoretical median DNA fragment length (1/*λ*, bp) and cytosine deamination rates observed as C→T misincorporations at 5′ read termini (MQ ≥ 30) by burial context. Pairwise comparisons with statistically significant differences (*t*-test; *p* < 0.01) are shown at the top of each plot. Comparisons for postcranial bones were not plotted, given that no comparisons had more than 5 data points.

### 3.4. Other factors affecting DNA preservation

Beyond sample age, sample type and burial context, a range of additional variables can influence DNA preservation. Neutral to slightly alkaline soils are generally more favourable [20,30], because higher pH slows hydrolytic DNA decay and often reduces microbial activity—two major drivers of molecular degradation. Although we lacked soil pH data to test this directly, the underlying geology appears to correlate with DNA recovery. In the Lesser Antilles and Aruba, for example, volcanic soils tend to be more acidic (typically pH < 6) [61], and DNA recovery has so far proven particularly difficult. By contrast, limestone bedrock and associated sediments, which typically maintain a higher pH (approx. 7–9), appear to provide more favourable conditions for DNA preservation. The limestone rock shelter of Canímar Abajo in Cuba exemplifies this pattern, yielding not only some of the best-preserved samples in our dataset but also some of the oldest human DNA recovered in the Caribbean to date [5].

Although we were unable to test them directly in the present study, several additional environmental and post-depositional factors are also likely to influence DNA preservation and warrant investigation in future work. Soil organic matter, for instance, influences microbial activity and processes such as root penetration and eluviation, all of which accelerate degradation [30]. Burial depth and exposure also play a role: deeper burials are significantly more likely to retain recoverable DNA, whereas surface weathering and water flow hasten DNA loss [62,63]. Finally, post-excavation treatment and storage can have substantial effects; Pruvost *et al.* [64], for example, showed that freshly excavated, unwashed bones contained roughly six times more amplifiable DNA than washed bones stored at room temperature. Incorporating such variables into future studies could help explain additional variation in DNA preservation beyond that accounted for by sample type, burial context, and age.

Beyond environmental and post-depositional factors, methodological differences may also affect DNA recovery. To explore this, we evaluated the effects of different DNA extraction and library preparation protocols while accounting for tissue type and burial context (supplementary material, note 3). In most cases, we found no significant differences among protocols in endogenous DNA recovery, DNA damage or fragmentation (*p* > 0.01). The only exception was petrous bones from open sites, for which we found significant differences in endogenous DNA content, DNA damage and fragmentation between some extraction protocols and between the two library preparation methods (supplementary material, table S4, note 3). However, because all samples from a given site were processed using the same protocols and the relevant subsets were small, we cannot exclude the possibility that the observed differences between protocols partly reflect underlying variation in preservation among sites.

Regardless of the underlying causes, DNA preservation varied markedly across our dataset. Among the petrous bones, endogenous DNA ranged from 0.01 to 66.5%, theoretical median fragment lengths from 8 to 63 bp, and 5′ deamination rates from 10 to 49%, with similarly large variation even within single sites such as Canímar Abajo (supplementary material, figure S2). This degree of variation indicates that screening one or two individuals from a site does not provide a reliable picture of overall preservation. Instead, identifying well-preserved material generally requires screening multiple individuals, and visual inspection can help prioritize samples that are more likely to yield good results. Because the analyses are destructive, such screening must be balanced with ethical considerations, planned in consultation with local communities and stakeholders and designed to be as targeted and minimally invasive as possible [28,65].

### 3.5. DNA decay rates and maximum half-life

While our cross-sectional comparisons revealed no simple correlation between sample age and preservation, estimating DNA decay rates provides a way to integrate temporal information and compare degradation across environments. Using 75 radiocarbon-dated Caribbean samples and 24 published genomes from North America, including Greenland and Alaska [46–52], we estimate that DNA decays on average approximately 4× faster in the Caribbean than in temperate North America and approximately 14× faster than in subpolar Greenland and Alaska (supplementary material, table S5). This difference remains largely consistent when considering only open sites, for which multiple samples are available (supplementary material, table S5). Using the slowest-decaying Caribbean sample (CAB_119) as a benchmark, we estimate that >99.9% of fragments would fall below 30 bp after approximately 5300 years, rendering them unmappable [66]. We conclude that recovery of aDNA from skeletal material older than approximately 5000 years in the Caribbean will be extremely challenging. However, as this period covers most of the region's human history, there is still significant scope for aDNA to address long-standing questions about origins, interactions and demographic change in the region.

### 3.6. Target enrichment of human DNA (1240k capture)

We evaluated the performance of 1240k capture in 82 Caribbean samples by comparing shotgun with previously published capture data [5] (supplementary material, table S3). On average, enrichment increased recovery at targeted sites 280-fold (range: 1.5–7268; excluding one outlier of 12 978), with no significant differences across sample types or burial contexts. Efficiency was inversely correlated with endogenous DNA content: samples with <5% endogenous DNA averaged 606-fold enrichment (median = 138), while those with >5% showed only approximately 12-fold gains (median = 11) (supplementary material, figure S3). Thus, enrichment is particularly valuable for poorly preserved samples but provides limited benefit for those with better preservation. However, as observed previously [67,68], enrichment also increased clonality and shifted GC content from 42.5% in shotgun libraries to 55.1% in captured libraries, likely reflecting hybridization bias which can complicate combined analyses of shotgun and capture data [69]. Nonetheless, enrichment remains a powerful—and often the only feasible—strategy for generating sufficient data from poorly preserved samples for population genetics.

## 4. Conclusions

This study provides the first systematic characterization of where, how and for how long aDNA is preserved in the Caribbean. We show that DNA decays significantly faster in tropical environments than in temperate or subpolar regions, and our decay-rate estimates suggest that recovery from skeletal remains older than approximately 5000 years will be extremely difficult. While this time frame encompasses much of the region's human history, it places realistic limits on what can be achieved.

Preservation is also highly variable, both between and within sites, making outcomes difficult to predict and underscoring the importance of systematic screening and careful sampling strategies. Realistically, genome-wide target enrichment will often be necessary to obtain usable data, though researchers should be aware that this can introduce biases that must be taken into account [69]. Ethically, the destructive nature of aDNA analysis makes it essential to minimize unnecessary sampling and to report both successes and failures to build a more complete, region-wide picture of DNA preservation. Equally important is that limitations and expectations are being discussed with local communities and stakeholders, so that informed decisions on sampling strategy can be developed together, ensuring that research priorities and practices align with their expectations and values [70].

Based on these observations, several practical lessons emerge:

— *Expect faster decay:* DNA survival is limited compared to temperate/subpolar regions, with recovery unlikely beyond approximately 5000 years.
— *Use visual inspection:* While imperfect, macroscopic assessment is a useful first line triage method to reduce unnecessary destructive analysis.
— *Screen systematically:* Large intra- and inter-site variation means that preservation cannot be assumed even within a single context.
— *Prioritize sample choice:* Petrous bones and teeth (especially cementum) are most promising; rib and long bone fragments should generally be avoided.
— *Consider burial context:* Cave and sheltered sites may yield better-preserved remains, but multiple samples per site may be needed.
— *Consider geology and soil chemistry:* Limestone bedrock and neutral–alkaline sediments are generally more favourable for DNA preservation than volcanic or iron-rich, more acidic soils.
— *Apply enrichment judiciously:* Target capture can rescue poorly preserved samples but introduces biases that complicate comparisons with shotgun data.
— *Report comprehensively:* Including negative results is essential for refining preservation expectations and guiding future projects.
— *Plan for poor preservation:* Because only a minority of samples may be amenable to shotgun sequencing, projects should budget for extensive screening and incorporate fallback strategies such as target enrichment.
— *Engage ethically:* Because aDNA work is destructive, sampling should be planned in consultation with local communities and stakeholders and designed to balance scientific aims with cultural and ethical responsibilities.

## Supplementary Material



## Data Availability

The new sequencing data generated for this paper can be found in the European Nucleotide Archive (ENA) under project PRJEB75577. Supplementary material is available online [70].
