# Supplementary material for: Ancient DNA preservation in the Caribbean
Source: R Soc Open Sci. 2026 Sep 9;13(9):260217. doi: 10.1098/rsos.260217 (PMC13698031; doi:10.1098/rsos.260217)
Supplement: Supplementary file 1 [file rsos.260217.zip › rsos.260217/rsos260217_si_001.pdf]

Supplemental Material

Supplementary Note 1. Sample acquisition and permissions

The archaeological human remains analysed in this study derive from 33 archaeological sites across the Caribbean, dating to ~3,200-400 cal BP. Samples were collected over a period of more than two decades (1998-2020) through multiple field projects, rescue excavations, and museum collaborations across various islands and jurisdictions, and incorporating data from both published studies and ongoing projects.

Because the Caribbean is a region with diverse political structures, legal frameworks, and heritage-management traditions, the permitting process necessarily varied from island to island [1]. Each territory has its own governmental agencies, archaeological oversight bodies, and institutional procedures governing access to human remains. Permissions to conduct destructive analyses, including ancient DNA extraction and radiocarbon dating, were obtained from the relevant government authorities, museums, or collaborating institutions responsible for collections care and cultural heritage management (see Supplementary Table S1).

In keeping with current best practice in ancient DNA research [1,2], sampling decisions for the original studies [e.g. 3,4–6] – each with its own aims and research questions – were made in consultation with archaeologists, museum curators, heritage managers, and local communities and community representatives familiar with the cultural, historical, and social context of the remains. However, in many parts of the Caribbean, the identification and formal recognition of descendant or Indigenous communities is complex and uneven. While Indigenous ancestry persists across the region to varying degrees [4], legally recognised descendant communities are limited, and in some cases absent, particularly compared to other world regions.

Within this context, engagement often took place with local communities, cultural organisations, and stakeholder groups who, while not necessarily direct descendants of the individuals studied, nonetheless have longstanding historical, cultural, and ethical interests in Caribbean archaeological heritage. This included, for example, collaboration and dialogue with descendant communities in Dominica and St Vincent (e.g. through the Nexus 1492 framework), as well as engagement with cultural groups and descendant communities in the Dominican Republic, Puerto Rico, and the wider diaspora. These discussions informed not only the selection of individuals for analysis but also the scope of laboratory work, the degree of destructive sampling permitted, and the sharing of results.

The present meta-study builds on these earlier consultations to evaluate outcomes across projects and to help inform future sampling strategies. Given the destructive nature of aDNA analysis, we took several measures to minimise unnecessary impact on cultural heritage. All samples were visually assessed for macroscopic preservation prior to destructive analysis; where possible, we prioritised skeletal elements known to yield higher endogenous DNA (e.g., petrous bones, tooth cementum) to reduce the number of samples required. Across all sites, we adhered to established protocols for minimal-impact sampling and ensured that all unused material was returned to the curating institution after analysis.

Supplementary Note 2. Data generation

Samples were processed in three ancient DNA laboratories using three different DNA extraction protocols and library preparation strategies (Table S1). All work followed strict aDNA standards to minimise contamination, including dedicated clean rooms, protective clothing, and negative controls. Together, these protocols and sequencing platforms introduce potential batch effects, which we later tested for but did not detect (see Results and Supplementary Materials).

2.1 DNA extraction

Samples from the Bahamas, Dominican Republic, Guadeloupe, Aruba, Cuba, Saba, St. Lucia, and Trinidad and Tobago were processed at the University of Copenhagen using a silica-in-solution protocol with a 15-minute pre-digestion step [7]. Samples were cleaned with a bleach wipe before DNA extraction. Tooth roots were separated using a Dremel rotatory tool and cementum was targeted whenever possible [7]. The petrous bones were sampled by isolating the cochlea [8]. Subsequently, c. 100-200 mg of root/bone powder following a modified silica-in-solution protocol, which includes a brief pre-digestion step [7]. Briefly, 1 ml of 0.45 M EDTA and 0.25 mg/ml proteinase K, pH 8.0 was added to all samples and incubated for 10 minutes at 37°C. After centrifuging the samples at 2000 rpm for 1 minute and removing the supernatant, the bone powder was resuspended in 4 ml of the same digestion buffer and incubated on a rotor overnight at 37°C. The next day, the samples were again centrifuged at 2000 rpm for 1 minute, and the supernatant was transferred to 40 ml of binding buffer (pH = 4) consisting of 5 M GuHCl, 20 mM NaCl, 100 mM NaOAc and 30% isopropanol [9]. After adding a silica suspension (50 µl), the pH was adjusted to be between 4.0-5.0, and the samples were incubated on a rotor at room temperature for 1 hour. The samples were then spun down for 5 minutes at 2000 rpm, and the supernatant was removed. After two washes with 1 ml 80% EtOH, the silica pellets were left to dry. For the elution, 64 µl of EB was added to the dried silica pellets, incubated for 10 minutes in a shaker at 37°C, spun down, and the supernatant transferred to new 1.5 ml DNA Lo-bind Eppendorf tubes.

Samples from Anse à la Gourde, Guadeloupe, were processed at the Max Planck Institute for the Science of Human History in Jena, Germany, using c. 100 mg of bone powder and a solution of 900 µl EDTA, 75 µl H2O and 25 µl Proteinase K. They were digested for at least 16h at 37°C, followed by an additional hour at 56°C as previously described in [10]. The solution was centrifuged, transferred into a binding buffer and purified using silica columns from High Pure Viral Nucleic Acid Large Volume Kit (Roche) as described in [11]. After washing the columns twice using the manufacturer's wash buffer, DNA was finally eluted in 100 µl of 6/39 TET (10 mM Tris, 1 mM EDTA and 0.05% Tween). The samples from Paso del Indio in Puerto Rico were processed at the ancient DNA laboratory at the Arizona State University, Tempe, AZ, following similar protocols [12] and using c. 50 mg of starting material.

2.2 Library preparation and sequencing

Samples from the Bahamas, the Dominican Republic, Guadeloupe and the site of El Chorro de Maíta in Cuba, processed in Copenhagen, were built into Illumina sequencing libraries using a double-stranded protocol [13]. For the end-repair step, 30 µl of DNA extract was added to a 50 µl reaction, incubated for 20 min at 12°C and 15 min at 37°C, purified with Qiagen MinElute columns, and eluted in 30 µl. Adapter ligation was performed in 50 µl reactions with Illumina-specific adapters, incubated for 15 min at 20°C, purified with Qiagen QiaQuick columns, and eluted in 30 µl EB. Adapter fill-in was carried out in 50 µl reactions at 37°C for 20 min, followed by 80°C for 20 min. Libraries were amplified in 50 µl PCR reactions containing 10 µl library template, 25 µl KAPA HiFi HotStart Uracil+ mix, 1.5 µl DMSO, 1 µl BSA (20

mg/ml), and 1 µl each of P5 and P7 dual-indexing primers (10 µM) [13]. Cycling conditions were 98°C for 5 min, 10–20 cycles of 98°C for 15 s, 60°C for 20 s, and 72°C for 20 s, with a final 72°C extension for 1 min. The optimal cycle number was determined by qPCR. Amplified libraries were purified with AMPure XP beads, eluted in 30 µl EB, quantified on an Agilent TapeStation, pooled in equimolar amounts, and sequenced as single-end 80–100 cycles on an Illumina HiSeq2500.

The remaining samples from Aruba, Cuba, Saba, St. Lucia, and Trinidad and Tobago processed in Copenhagen were built into Illumina libraries using an optimised single-tube protocol [14]. End-repair was performed in 40 µl reactions with 32 µl DNA extract, incubated 30 min at 20°C and 30 min at 65°C. Adapter ligation was carried out with 2 µl adapters and T4 ligase in 50 µl reactions for 30 min at 20°C and 10 min at 65°C. Fill-in was performed by adding 10 µl master mix, incubating 15 min at 65°C and 15 min at 80°C. Libraries were purified with SPRI magnetic beads, eluted in 60 µl EB, amplified, dual-indexed [13], quantified, pooled, and sequenced single-end (80 cycles) on HiSeq2500/4000.

Ten samples from Paso del Indio (Puerto Rico) were processed at Arizona State University using the same double-stranded protocol [15] but with different PCR setups. Four 100 µl indexing reactions per library were performed, each containing 10 µl Pfu Turbo buffer, 2.5 µl dNTPs (10 mM), 1.5 µl BSA (10 mg/ml), 2 µl each of P5 and P7 primers (10 µM), 1 µl Pfu Turbo enzyme, and 9 µl DNA library template. PCR cycling was 95°C for 15 min, 10 cycles of 95°C for 30 s, 58°C for 30 s, and 72°C for 45 s, with a final 72°C for 10 min. Products were purified with Qiagen MinElute columns using preheated EB (65°C) and eluted in 30 µl. Libraries underwent a second amplification round with four 100 µl reactions containing 10 µl Accuprime Pfx buffer, 3 µl IS5 primer (10 µM), 3 µl IS6 primer (10 µM), 76 µl H_2O, 1 µl Accuprime Pfx enzyme, and 7 µl template. Cycling was 95°C for 2 min, 7–13 cycles of 95°C for 15 s, 60°C for 30 s, and 68°C for 1 min, followed by 68°C for 5 min. Products were purified, quantified on an Agilent Bioanalyzer, and sequenced paired-end with 75 cycles on an Illumina NextSeq500.

Samples from Cuba and Puerto Rico processed at the Max Planck Institute (Jena) were prepared with a double-stranded protocol, following partial UDG treatment [13]. Extracts (25 µl) were mixed with 6 µl 10× Tango buffer, 0.2 µl dNTPs (25 mM), 6 µl ATP (100 mM), 0.5 µl BSA (20 mg/ml), 3.6 µl USER enzyme mix, and 8.7 µl HPLC-H_2O, incubated 30 min at 37°C and 1 min at 12°C. UGI (3.6 µl, 2 U/µl) was added, incubated 30 min at 37°C and 1 min at 12°C. Blunt-end repair used 3 µl T4 kinase and 1.65 µl T4 polymerase at 25°C for 20 min and 12°C for 10 min. Products were purified with MinElute (650 µl PB binding buffer, 700 µl PE wash, eluted in 20 µl preheated EBT). Adapter ligation was carried out in 40 µl with 20 µl Quick Ligase buffer, 1 µl adapters (10 µM), and 1 µl Quick Ligase, incubated at 22°C for 20 min. Fill-in was performed with 4 µl Isothermal buffer, 0.2 µl dNTPs (25 mM), 2 µl Bst polymerase, 13.8 µl H_2O, and 20 µl eluate at 37°C for 30 min and 80°C for 10 min. Libraries were amplified and purified as described above using dual indexes [13], and sequenced single- or paired-end (50–75 cycles) on a HiSeq4000.

Finally, a subset of samples from Anse à la Gourde (Guadeloupe) and Puerto Rico processed at the MPI Leipzig used an automated single-stranded protocol with partial UDG treatment [16]. DNA extracts (30 µl) were incubated with ligation buffer, Tween-20, and Fast AP, then ligated to adapters with PEG-8000, ATP, and T4 ligase. Products were immobilised with Dynabeads, washed, and a second strand synthesised with Klenow buffer, dNTPs, adapters, and Klenow polymerase. After another bead wash and a second ligation, libraries were amplified with AccuPrime Pfx polymerase and double indexes. Products were sequenced with 75 cycles on an Illumina HiSeq4000 in single-read mode.

Supplementary Note 3. Assessing potential library and platform biases

To test for possible batch effects, we compared the human DNA endogenous content, theoretical DNA fragment length, and ancient DNA damage (% C→T misincorporations at 5' termini) across datasets generated with different DNA extraction protocols [7,10,12], library preparation protocols [13,14,16], library types (single-stranded DNA or double-stranded DNA) and sequencing platforms (NextSeq500, HiSeq2500 and HiSeq4000). For each of the preservation metrics, we tested if it was statistically significant between different datasets using a two-sample Student's t-tests. For each test, we considered samples from different types (petrous bones, teeth and post-cranial bones) and burial contexts (open and cave) separately, and only comparisons with at least 5 data points were considered. For most of the comparisons, we do not find statistically significant differences (t-test: $p > 0.01$). Below we describe the significant differences that we obtained.

Finally, when the number of samples per library preparation protocol or sequencing platform allowed it, we tested whether the differences observed in human DNA endogenous content, theoretical DNA fragment length, and ancient DNA damage in the complete dataset, remained statistically significant when restricting to samples from a single DNA extraction method, library preparation protocol, library type or sequencing platform. All the differences observed in the complete dataset can be reproduced when restricting to a single extraction protocol, library preparation protocol or sequencing platform.

DNA extraction protocol. Significant differences across DNA extraction protocols were exclusively found within petrous bones from open sites. Samples extracted with the Rohland & Hofreiter (2007) protocol had higher human DNA endogenous content compared to Dabney & Meyer (2019) and to Damgaard et al. (2015) protocols. DNA was significantly more fragmented but less damaged in samples extracted with the Dabney & Meyer (2019) protocol compared to the other two protocols.

Library preparation protocol. Significant differences between library preparation protocols were exclusively found within petrous bones from open sites. Libraries prepared with the Carøe et al. (2018) protocol had a significantly lower proportion of human endogenous DNA but higher DNA damage compared to those prepared with the Kircher et al. (2012) protocol.

Library type. We found no significant differences in any of the three preservation metrics between single- and double-stranded libraries.

Sequencing platform. For petrous bones from open sites, we find that sequencing data generated with the HiSeq2500 platform had significantly higher DNA damage compared to that generated with the HiSeq4000 platform.

Supplementary Note 4: Multivariate analyses of DNA preservation.
To assess whether the main patterns observed in the univariate analyses are robust when considering multiple variables simultaneously, we performed multivariate linear regression analyses using three response variables: (i) endogenous DNA content (%Endo), (ii) cytosine deamination rates (%C→T at 5′ termini), and (iii) theoretical fragment length (1/λ). As explanatory variables, we included burial context (open site, cave, rock shelter), sample type (petrous bone, tooth, postcranial bone), and sample age (radiocarbon age in cal BP, where available). All non-factor variables were log-transformed prior to analysis.

Dataset structure and limitations
The dataset is highly unbalanced with respect to both burial context and sample type. Of the 318 samples included in the study, 230 derive from open sites, 49 from caves, and 32 from rock shelters. Similarly, the distribution of sample types is uneven, with 217 teeth, 88 petrous bones, and 13 postcranial elements. The joint distribution of these variables is also sparse in places, with some combinations poorly represented or absent (e.g. no postcranial elements from rock shelters):

	Cave	Open	Rock shelter
Petrous bone	17	47	17
Postcranial bone	8	5	0
Tooth	24	178	15

In addition, key variables are only available for subsets of the data. Radiocarbon ages are available for 103 of 318 samples, while fragment length estimates (1/λ) are available for 115 samples, with only 76 samples having both age and fragment length estimates. As a result, multivariate models including age are based on substantially reduced sample sizes.

General observations
The unbalanced structure of the dataset has important implications for model interpretation. Because the majority of samples derive from open contexts, any robust effect of sample type or age would be expected to be detectable within this subset alone. However, as shown below, this is generally not the case. In addition, inclusion of sample age as a covariate further reduces sample size and can influence the apparent significance of other variables. Model diagnostics based on residual distributions (including QQ-plots and leverage statistics) were generally poor, particularly for deamination rates and fragment length, indicating deviations from standard model assumptions and limiting confidence in parameter estimates.

Deamination rates (%C→T)
Regression of deamination rates on burial context, sample type, and sample age yielded a low explanatory power (multiple $R^2 < 0.10$), both with and without inclusion of age. Sample age was not a significant predictor. The significance of burial context and sample type varied depending on model specification, and no consistent pattern was observed. Given the low explanatory power and poor model diagnostics, further analysis of this response variable was not pursued.

Endogenous DNA content (%Endo)
Regression of endogenous DNA content on burial context, sample type, and sample age yielded a multiple R^2 of approximately 0.5. In this model, both sample type and burial context were significant predictors, whereas sample age was not. Excluding sample age from the model reduced the R^2 to approximately 0.3 but did not change the significance of sample type and

burial context. However, when the analysis was restricted to samples from open contexts (which constitute the majority of the dataset), the significance of both sample type and burial context disappeared, and model fit was reduced. This suggests that the apparent effects of these variables are sensitive to the uneven structure of the dataset and may partly reflect confounding between context, sample composition, and preservation conditions.

Fragment length (1/λ)

Regression of theoretical fragment length (1/λ) on burial context, sample type, and sample age yielded a multiple R^2 of approximately 0.4, with sample age identified as a significant predictor. However, when restricting the analysis to samples from open contexts, neither sample type nor burial context remained significant, and the R^2 dropped below 0.15. As with endogenous DNA content, these findings indicate that the results are not robust across subsets of the data.

Summary

Across all three response variables, the multivariate analyses were limited by the highly unbalanced and incomplete nature of the dataset. Although some effects (notably of sample type and burial context on endogenous DNA content) were detected, these were not consistently reproducible across subsets of the data and were sensitive to model specification. In addition, model diagnostics were often poor, further limiting confidence in the results. Taken together, these findings suggest that, for this dataset, multivariate models do not provide robust additional insights beyond the univariate analyses presented in the main text. Instead, the simpler univariate framework offers a more transparent and reliable basis for interpreting patterns of DNA preservation.

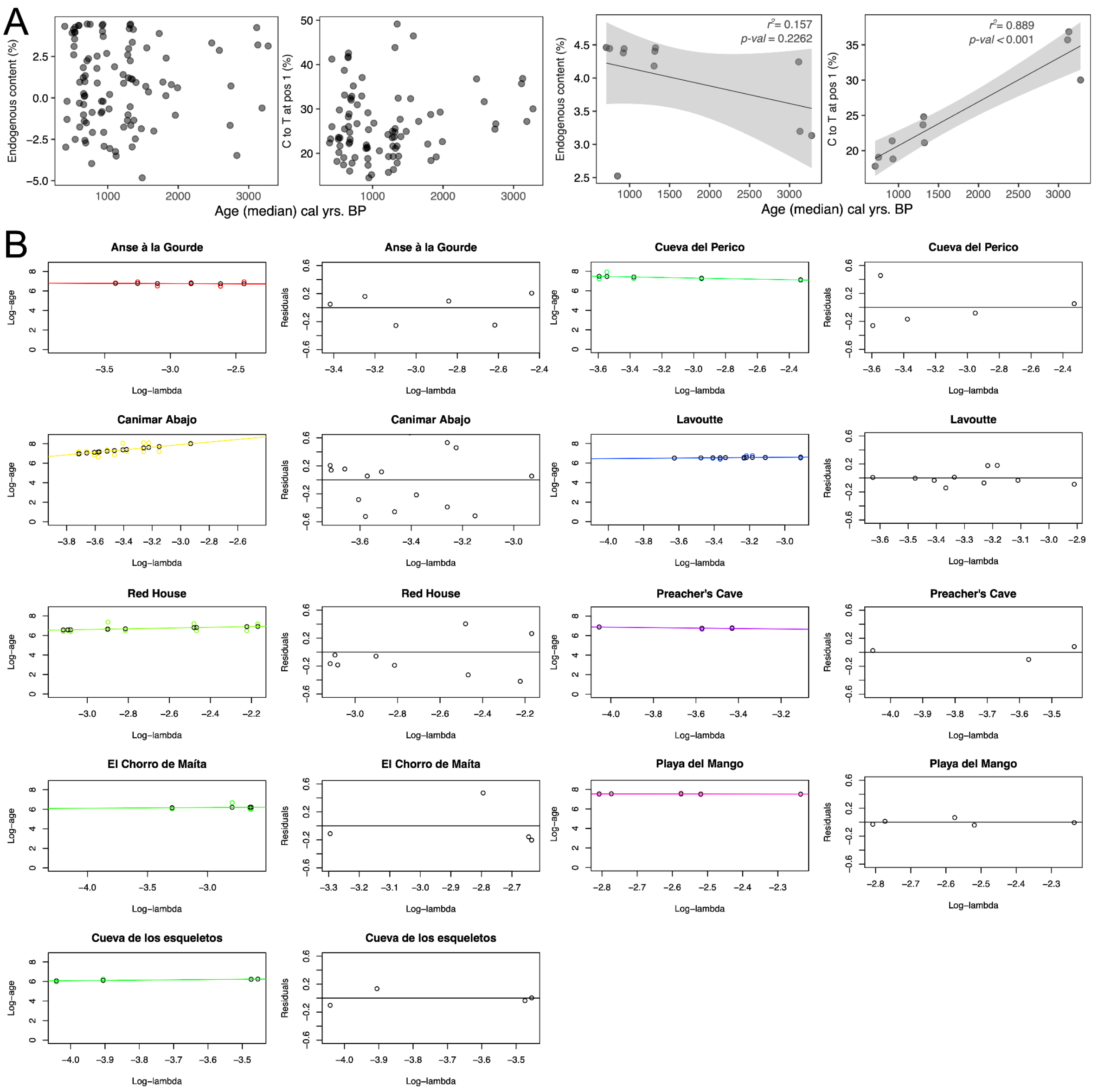

Figure S1. A. Human endogenous DNA content estimated before removing PCR duplicates (%, displayed in *log* scale) and C→T misincorporations at 5′ read termini (MQ ≥30, %) for 102 directly dated human skeletal samples from 22 archaeological sites in the Caribbean (top row) and 11 petrous bones from the site of Canimar Abajo in Cuba only fit with a linear regression model (bottom row). Grey line and shaded area indicate the fitted line and confidence intervals, respectively, and the resulting r^2 and p-values are shown in each case. **B.** Linear regression and residuals modeling the log-age vs log-lambda for each of the sites with at least 4 observations and radiocarbon dates for at least 1 sample.

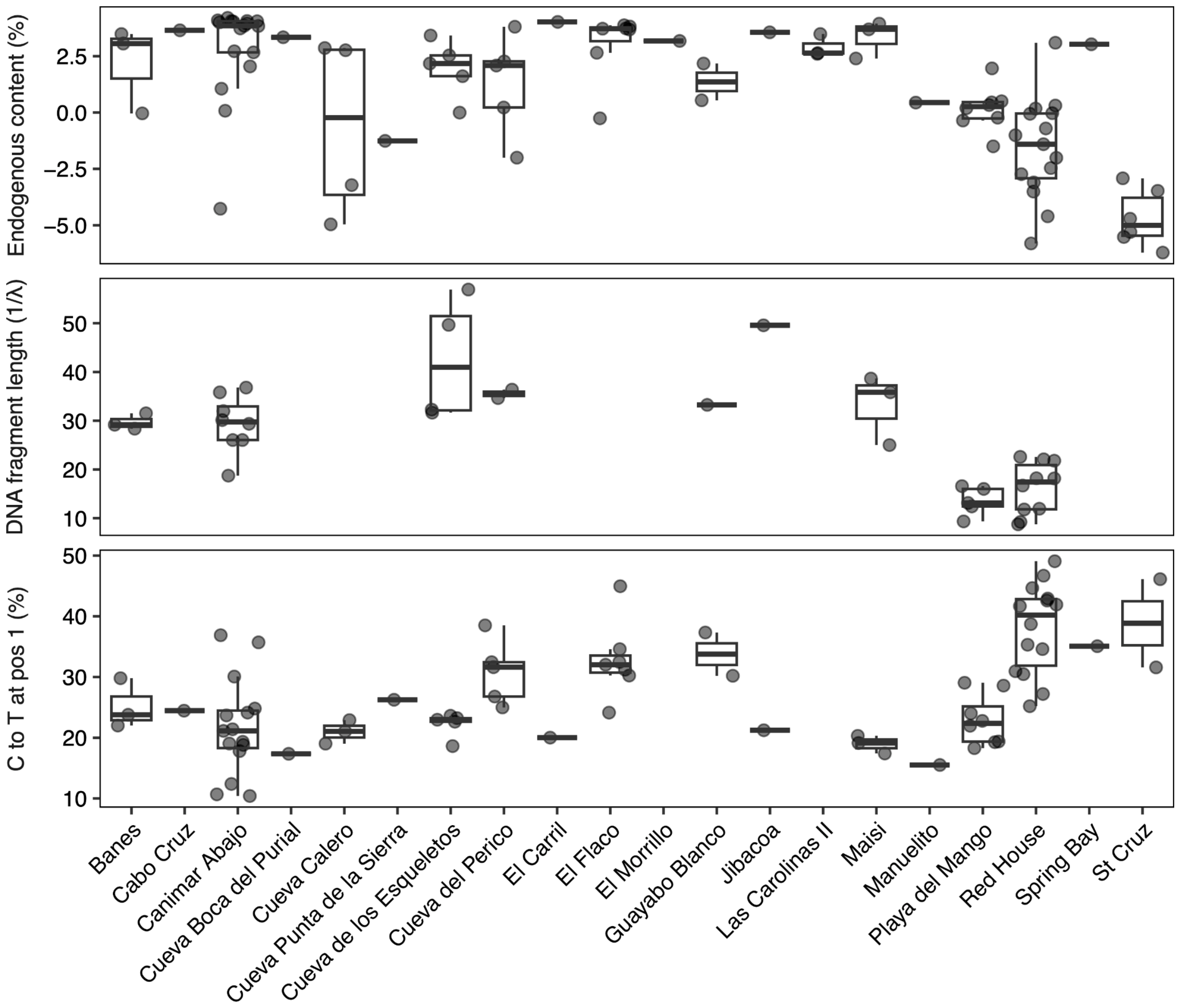

Figure S2. Within-site variation in DNA preservation. Human endogenous DNA content (%, displayed in *log* scale), theoretical DNA fragment length (1/λ, bp), and C→T misincorporations at 5′ read termini (MQ ≥30, %) for 74 petrous bones listed by site. Banes and Maisi are municipalities in Cuba, and the exact location of the samples is unknown.

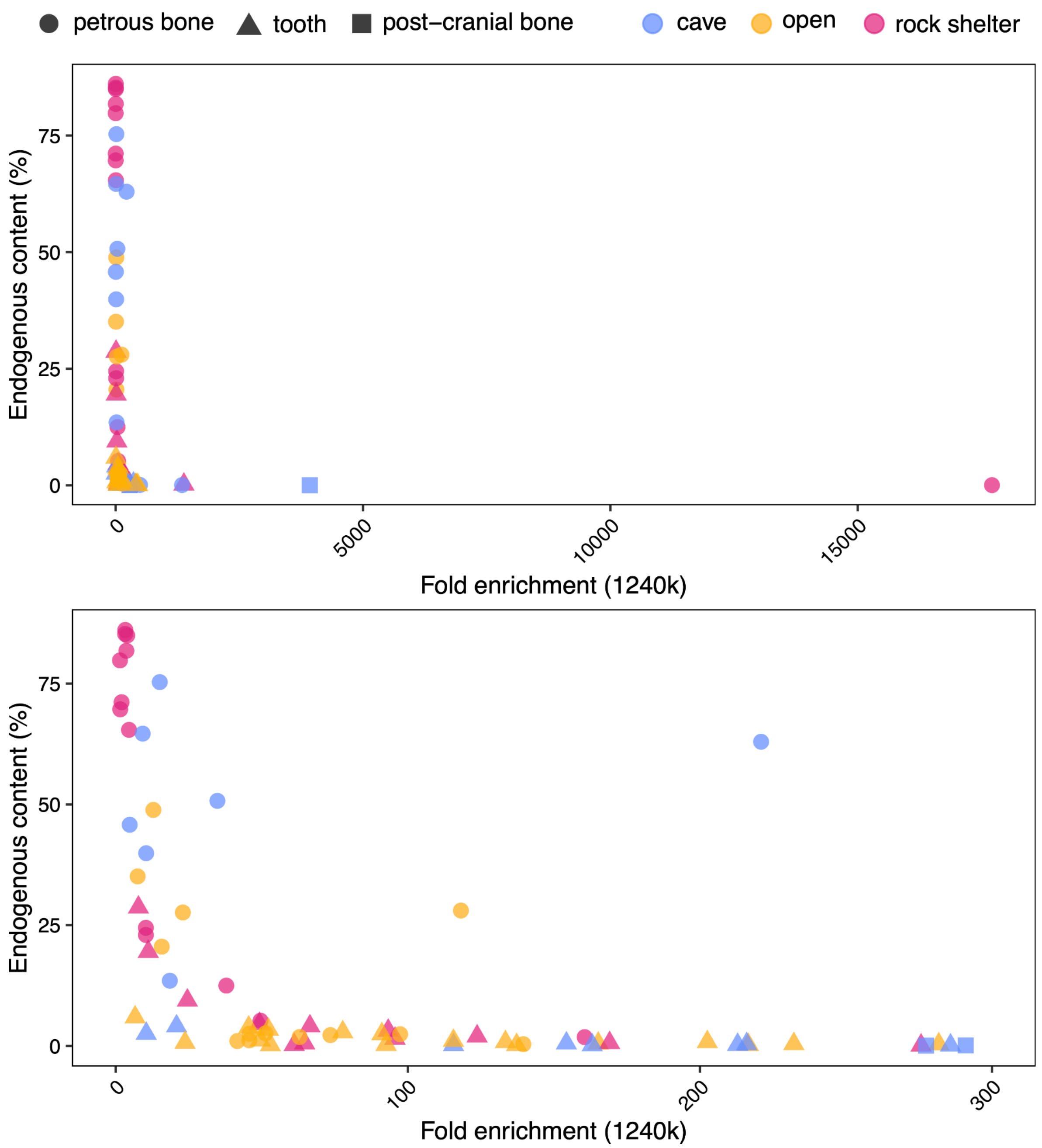

Figure S3. Fold enrichment on the 1240K capture assay for a subset of 82 samples (top) and after excluding outliers with >300 fold-enrichment (bottom). The percentage of endogenous DNA content was estimated after PCR duplicate removal. Sample types are indicated with different shapes, and burial context with different colours.

Supplementary references

1. Nägele K, Benn Torres J, Nieves-Colón MA. 2026 Research Ethics in Caribbean Archaeogenomics. In *Ethics in Caribbean Archaeology* (eds FJ Fricke, E Herrera Malatesta, MS de Waal), Gainesville, FL: University Press of Florida.

2. Bardill J, Bader AC, Garrison NA, Bolnick DA, Raff JA, Walker A, Malhi RS, Summer internship for INdigenous peoples in Genomics (SING) Consortium. 2018 Advancing

the ethics of paleogenomics. *Science* **360**, 384–385.

3. Schroeder H *et al.* 2015 Genome-wide ancestry of 17th-century enslaved Africans from the Caribbean. *Proc. Natl. Acad. Sci. U. S. A.* **112**, 3669–3673.

4. Schroeder H *et al.* 2018 Origins and genetic legacies of the Caribbean Taino. *Proc. Natl. Acad. Sci. U. S. A.* **115**, 2341–2346.

5. Nägele K *et al.* 2020 Genomic insights into the early peopling of the Caribbean. *Science* **369**, 456–460.

6. Nieves-Colón MA *et al.* 2020 Ancient DNA Reconstructs the Genetic Legacies of Precontact Puerto Rico Communities. *Mol. Biol. Evol.* **37**, 611–626.

7. Damgaard PB, Margaryan A, Schroeder H, Orlando L, Willerslev E, Allentoft ME. 2015 Improving access to endogenous DNA in ancient bones and teeth. *Sci. Rep.* **5**, 11184.

8. Pinhasi R *et al.* 2015 Optimal Ancient DNA Yields from the Inner Ear Part of the Human Petrous Bone. *PLoS One* **10**, e0129102.

9. Allentoft ME *et al.* 2015 Population genomics of Bronze Age Eurasia. *Nature* **522**, 167–172.

10. Rohland N, Hofreiter M. 2007 Ancient DNA extraction from bones and teeth. *Nat. Protoc.* **2**, 1756–1762.

11. Dabney J *et al.* 2013 Complete mitochondrial genome sequence of a Middle Pleistocene cave bear reconstructed from ultrashort DNA fragments. *Proceedings of the National Academy of Sciences* **110**, 15758–15763.

12. Dabney J, Meyer M. 2019 Extraction of highly degraded DNA from ancient bones and teeth. *Methods Mol. Biol.* **1963**, 25–29.

13. Kircher M, Sawyer S, Meyer M. 2012 Double indexing overcomes inaccuracies in multiplex sequencing on the Illumina platform. *Nucleic Acids Res.* **40**, e3.

14. Carøe C, Gopalakrishnan S, Vinner L, Mak S, Sinding M, Samaniego J, Wales N, Sicheritz-Pontén T, Gilbert T. 2018 Single-tube library preparation for degraded DNA. *Methods Ecol. Evol.* **9**, 410–419.

15. Meyer M, Kircher M. 2010 Illumina sequencing library preparation for highly multiplexed target capture and sequencing. *Cold Spring Harb. Protoc.* **2010**, db.prot5448.

16. Gansauge M-T, Aximu-Petri A, Nagel S, Meyer M. 2020 Manual and automated preparation of single-stranded DNA libraries for the sequencing of DNA from ancient biological remains and other sources of highly degraded DNA. *Nature Protocols*. **15**, 2279–2300. (doi:10.1038/s41596-020-0338-0)

17. Wales N, Carøe C, Sandoval-Velasco M, Gamba C, Barnett R, Samaniego JA, Madrigal JR, Orlando L, Gilbert MTP. 2015 New insights on single-stranded versus double-stranded DNA library preparation for ancient DNA. *Biotechniques* **59**, 368–371.
